# Peptides in Dentistry: A Scoping Review

**DOI:** 10.3390/bioengineering10020214

**Published:** 2023-02-06

**Authors:** Louis Hardan, Jean Claude Abou Chedid, Rim Bourgi, Carlos Enrique Cuevas-Suárez, Monika Lukomska-Szymanska, Vincenzo Tosco, Ana Josefina Monjarás-Ávila, Massa Jabra, Fouad Salloum-Yared, Naji Kharouf, Davide Mancino, Youssef Haikel

**Affiliations:** 1 Department of Restorative Dentistry, School of Dentistry, Saint Joseph University, Beirut 1107 2180, Lebanon; 2 Department of Pediatric Dentistry, Faculty of Dentistry, Saint Joseph University, Beirut 1107 2180, Lebanon; 3 Department of Biomaterials and Bioengineering, INSERM UMR_S 1121, University of Strasbourg, 67000 Strasbourg, France; 4 Dental Materials Laboratory, Academic Area of Dentistry, Autonomous University of Hidalgo State, San Agustín Tlaxiaca 42160, Mexico; 5 Department of General Dentistry, Medical University of Lodz, 251 Pomorska St., 92-213 Lodz, Poland; 6 Department of Clinical Sciences and Stomatology (DISCO), Polytechnic University of Marche, 60126 Ancona, Italy; 7 Faculty of Medicine, Damascus University, Damascus 0100, Syria; 8 Private Practice, 54290 Trier, Germany; 9 Department of Endodontics and Conservative Dentistry, Faculty of Dental Medicine, University of Strasbourg, 67000 Strasbourg, France; 10 Pôle de Médecine et Chirurgie Bucco-Dentaire, Hôpital Civil, Hôpitaux Universitaire de Strasbourg, 67000 Strasbourg, France

**Keywords:** antimicrobial, osseointegration, surface modification, tissue engineering

## Abstract

Currently, it remains unclear which specific peptides could be appropriate for applications in different fields of dentistry. The aim of this scoping review was to scan the contemporary scientific papers related to the types, uses and applications of peptides in dentistry at the moment. Literature database searches were performed in the following databases: PubMed/MEDLINE, Scopus, Web of Science, Embase, and Scielo. A total of 133 articles involving the use of peptides in dentistry-related applications were included. The studies involved experimental designs in animals, microorganisms, or cells; clinical trials were also identified within this review. Most of the applications of peptides included caries management, implant osseointegration, guided tissue regeneration, vital pulp therapy, antimicrobial activity, enamel remineralization, periodontal therapy, the surface modification of tooth implants, and the modification of other restorative materials such as dental adhesives and denture base resins. The in vitro and in vivo studies included in this review suggested that peptides may have beneficial effects for treating early carious lesions, promoting cell adhesion, enhancing the adhesion strength of dental implants, and in tissue engineering as healthy promotors of the periodontium and antimicrobial agents. The lack of clinical trials should be highlighted, leaving a wide space available for the investigation of peptides in dentistry.

## 1. Introduction

Dental plaques contain over 750 different bacterial species, which are the major reason for dental caries, with streptococci being the most predominantly present. These bacteria, due to the production of acids, can demineralize and affect mineralized tooth tissues [1]. Different additives and biomaterials were used in dental treatments in order to eliminate and decrease the number of bacteria in the oral cavity and teeth tissues. Some dental materials, such as calcium silicate-based products, have been introduced in the dental market due to their antibacterial, antioxidant and remineralization properties [2]. Other solutions that have antibacterial effects are used to clean the root canal and kill resistant bacteria in the root canal system [3]. Even though they sometimes display high cytotoxicity [4,5], these materials are still currently used in dentistry.

It should be remembered that a peptide is expressed as a short polymer of amino acids (AA) [6]. According to the description of diverse authors in the literature, sizes of peptides may vary from <20, <50, to <100 [7,8,9,10]. The use of peptides has been paid attention to over the last two decades [6,11]. These peptides were used in various dental fields such as in endodontic treatment, coronal restoration, caries management, bone and dental tissue remineralization and in the modification of dental materials in order to promote the biological effects of these materials in the oral environment [6,12].

In the last periods, over 7000 native peptides (NP) have been considered by means of significant human physiological functions [13]. These peptides have functions by way of cell-penetrating, cell adhesion motifs, tumor-homing peptides, neuropeptides, structural peptides, peptide hormones, antimicrobial peptides, peptide tags, matrix metalloprotease substrates, growth factors, amyloid peptides, and erstwhile diverse NPs [10].

Nevertheless, one should state that NPs are frequently not truthfully appropriate for therapeutic usage since they have intrinsic drawbacks, including their poor physical and chemical stability, low oral bioavailability, short flowing plasma half-life, and quick removal from the circulation through the kidneys and the liver [9,13,14]. It is well described that peptides such as insulin and adrenocorticotrophic hormone were used for human therapeutic purposes in the first half of the 20th century [15]. Later on, synthetic oxytocin and vasopressin arrived in clinical use in the 1950s with the chemical elucidation of the sequences of these peptides [16].

Lately, pharmaceutical manufacturing has amplified the consideration of novel therapeutic peptides, persistently touching clinical claims [9,17]. By 2018, more than 60 peptides were approved by the Food and Drug Administration (FDA), and more than 600 were undergoing preclinical and clinical examinations [9,18]. With the current elaborations of solid-phase peptide synthesis, the production of therapeutic synthetic peptides (SP) has become achievable [9]. Accordingly, innovative synthetic approaches permit the modulation of pharmacokinetic assets and focus on specificity through AAs, the integration of non-natural AAs, backbone adjustments, and the peptide conjugates refining solubility or prolonging the half-life [8,13,14].

It is recognized that human dental masses, once fashioned, cannot be biologically replaced or repaired, and their multifaceted conformations require diverse approaches for regeneration [6]. However, it is unclear in the literature which specific peptides could be effective for applications in different fields of dentistry. Thus, the aim of this scoping review was to map the contemporary scientific papers related to the use and applications of peptides in dentistry at present.

## 2. Materials and Methods

The present scoping review has been described according to the PRISMA extension for scoping reviews guideline [19]. The review protocol was registered at Open Science Framework, and it is available at https://osf.io/up6ty (accessed on 18 December 2022). The systematic search was performed according to the following parameters: (i) population: peer-reviewed articles; (ii) intervention: use of natural or synthetic peptides; (iii) comparison: other substances or treatments; (iv) outcome: dental applications, (v) study design: in vitro or in vivo articles. The general question of the review was as follows: what scientific applications of products based on peptides are being used for dental applications?

### 2.1. Information Sources and Search

The literature database search was performed by two independent reviewers (RB and CECS) until September 2022. The search was carried out in the following databases: PubMed/MEDLINE, Scopus, Web of Science, Embase, and Scielo. The search strategy was first defined for the MEDLINE database using a controlled vocabulary and free keywords (Table 1). The MEDLINE search strategy was then adapted to other electronic databases. The reviewers also hand-searched the reference lists of the included articles to identify additional manuscripts.

### 2.2. Selection Process and Data Collection Process

After running the search strategy, a reference management program was used (EndNote X9, Clarivate Analytics, Philadelphia, PA, USA) to store the files of all databases. Then, duplicate articles were removed, followed by manual removal after the organization of titles in alphabetical order. All studies were initially scanned for relevance by title followed by abstract using an online software program (Rayyan, Qatar Computing Research Institute, HBKU, Doha, Qatar). The titles and abstracts of the articles were screened according to the following inclusion criterium: in vitro or in vivo studies that evaluated or reported the use of peptides for dental applications. The search was carried out on documents published in any language without restrictions on their date of publication. Reviews, case reports, case series, pilot studies, and conference abstracts were excluded. If the review authors were not sure about the eligibility of any study, it was kept for the next phase. All phases were carried out by two independent reviewers (RB and CECS) to check whether they met the inclusion criteria. The same two reviewers summarized and categorized the data using a standardized form. The information collected included the type of study, the peptide used, the application proposed and the main results.

## 3. Results

**Figure 1:**
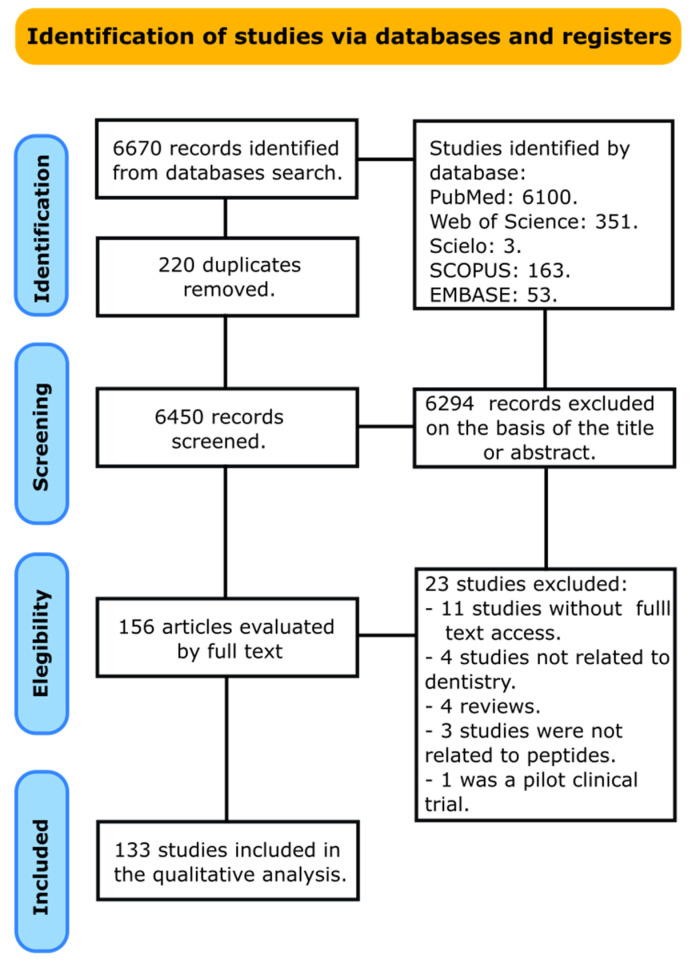
Flowchart summarizing the selection process of articles.

This scoping review is described according to the PRISMA extension for scoping reviews guideline [19]. After database screening and duplicate removal, a total of 6450 articles were recognized (Figure 1). After title and abstract screening, 156 articles remained for full-text inspection. From the 156 articles assessed for eligibility, 23 articles were excluded due to the following reasons: in 11 articles, the full text was not retrieved [20,21,22,23,24,25,26,27,28,29,30], 4 articles were not related to the dentistry field [31,32,33,34], 4 studies were reviews [6,12,35,36], 3 studies were not related to peptides [37,38,39], and 1 study was a pilot clinical trial [40]. Thus, a total of 133 articles were included in the present review.

### 3.1. Characteristics of Studies

The main characteristics of the studies included in the present review are presented in Table 2.

The studies included experiments in animals and/or using bacteria or cells; also, several clinical trials were found. Most of the applications of the peptides included caries management, implant osseointegration, guided tissue regeneration, vital pulp therapy, antimicrobial activity, enamel remineralization, occlusion of dentin tubules, periodontal therapy, the surface modification of dental implants, and the modification of dental materials such as dental adhesives and denture base resins.

### 3.2. Synthesis of Results and Summary of Evidence

The in vitro and in vivo studies included in the present review stated that peptides may have beneficial effects for treating early carious lesions. Additionally, the use of peptides seems to be beneficial for promoting cell adhesion and enhancing the adhesion strength of dental implants. In addition, peptides were useful for tissue engineering for cell-based pulp regeneration. Peptides were also successfully used as healthy promotors of the periodontium, acting as inflammatory mediators. Finally, most peptides were used as effective antimicrobial agents.

## 4. Discussion

A scoping review was performed regarding the use and applications of peptides in the dental field at present. Appropriately, most of the applications of the peptides included caries management, implant osseointegration, guided tissue regeneration, vital pulp therapy, antimicrobial activity, enamel remineralization, occlusion of dentin tubules, periodontal therapy, the surface modification of dental implants, and the modification of dental materials such as dental adhesives and denture base resins.

One should keep in mind that dental caries is considered the most common disease worldwide [171], and it can lead to the destruction of dental surfaces by means of acidogenic bacteria changing sugars to acids [43]. Dissolution of the mineral tooth structure begins with caries formation, therefore generating a demineralized subsurface lesion body, similar to white spots [172], followed by the development of irreversible cavitation of the mineralized surface layer [173,174]. Treatment of manifested caries involves an oral hygiene regulation and a follow-up visit to identify whether the caries has been prevented or has advanced into a cavity, which is subsequently treated by means of restoration [173]. The use of fluoride varnish can prevent caries formation by reinforcing the inorganic surface layer, consequently inhibiting the progression of caries [175,176,177]. Fluoride ions are preserved within the inorganic surface layer covering the demineralized carious lesion due to the high correspondence to hydroxyapatite [178]. Subsequently, the demineralized subsurface zone is not penetrated by fluoride; yet this is where remineralization would be essential in an attempt to regenerate decayed enamel tissue [43]. For this reason, novel methods for the treatment of caries have been introduced to mimic the structure of the enamel matrix, such as guided enamel regeneration (GER) [179].

It should be noted that self-assembling peptide (SAP) technology was designated on the reasonable design of a short hydrophilic peptide in combination with GER that builds into fibers, establishing a three-dimensional (3D) scaffold [180,181,182]. The surface features of the fibers might fluctuate, concurring with the physiological desires of the treated tissue [66,183]. This could be explained by the rational design criteria [183]. When treating early caries lesions, SAP P11-4 fibers have been adjusted to suitably bind ionic calcium and template hydroxyapatite formation, thus, accompanying remineralization in a comparable approach of amelogenin that supports the construction of the enamel. From this analysis, the SAP P11-4 fibers might be known as a biomimetic agent [66,74]. This could be in agreement with the finding of this review that demonstrated the potential effect of peptide P11-4 in caries management.

With regards to implant osseointegration, pure titanium is commercially used for implants in the dental field due to its possible resistance to corrosion, biocompatibility, and suitable mechanical properties [184,185,186]. Researchers have detected peri-implant bone resorption produced by peri-implantitis, which is considered the key reason for the failure of osseointegrated dental implants [187,188]. In this manner, surface modification of dental implants has been a topic of interest for researchers since titanium is an inert material that decreases the aptitude for remedial tissue therapy to succeed and resists bacterial settlement [189,190,191]. To counteract peri-implantitis and advance osseointegration, different type of coatings have been investigated [192]. Surfaces incorporating chlorhexidine, antimicrobial agents and antibiotics such as gentamicin, and surfaces incorporating chlorhexidine, poly-lysine, sliver, and chitosan have all been established for coating the titanium surface of implants [52]. However, some drawbacks could be noted with antibiotic-coated titanium, such as the controversial opinion about their bacterial resistance and host cytotoxicity [193]. In 2015, Zhou et al. demonstrated that antimicrobial peptides provided a promising bifunctional titanium surface and enhanced its bactericidal activity and cytocompatibility [168]. Likewise, a previous report suggested that after 6 weeks of implantation in rabbit femurs, titanium dental implants with an antimicrobial peptide GL13K coating allowed in vivo dental implant osseointegration at similar bone growth rates to gold-standard non-coated dental implants [52]. This could be explained by the fact that GL13K is bactericidal in solution against *Escherichia coli*, *Pseudomonas aeruginosa*, *Porphyromonas gingivalis* and *Streptococcus gordonii* [83,194,195]. Similarly, Yoshinari et al. proved that the antimicrobial and titanium-binding peptides were favorable for the diminution of biofilm formation on titanium surfaces [162]. In addition, a laminin-derived peptide was demonstrated to improve and enhance the integration of soft tissue on dental titanium implants [143]. Furthermore, an epithelial basement membrane was formed on a titanium surface when platelet-activating peptide was used [135]. All in all, this could clearly support the result of this review that the use of peptides seems to be beneficial for promoting cell adhesion and enhancing the adhesion strength of dental implants.

In addition, this analysis determined that peptides were useful for guided tissue regeneration [42]. This could be achieved when a combination of a synthetic peptide named P-15 (analog of collagen) and an anorganic bovine bone mineral (ABM) was used. ABM enhanced cell attachment by differentiation and cell binding, thus enhancing osseous formation and ensuing an accelerated periodontal ligament fibroblast attachment [109,196]. Adding to P-15, biocompatible and osteoconductive filler material was thus detected [42].

A major task in the use of tissue engineering for therapy in dentistry involves the initiation of tooth and bone regeneration. The dentin phosphophoryn-derived arginine-glycine-aspartic acid-containing peptide was demonstrated as a biodegradable, biocompatible, and bioactive material for dentin regeneration. These results could be clarified by the short AA sequences of the peptide used and by its 3D conformation essential for acquiring this function [46]. Accordingly, the peptide can be used in vital pulp therapy when a specific sequence is used.

Further, most peptides were used as effective antimicrobial agents. Peptide hydrogels have shown that ultrashort peptides (<8 amino acids) might self-assemble into hydrogels. These ultrashort peptides might be intended to integrate antimicrobial motifs, such as positively charged lysine residues; thus, the peptides have integral antimicrobial features [47]. The scheme and synthesis of biocompatible hydrogels with antimicrobial activity are of numerous interests for tissue engineering drives comprising the replacement of tissue in infected root canals [65,197,198]. Moreover, antimicrobial peptides were used in coated titanium surfaces [168], dental adhesives [147], caries infection [102], and plaque biofilm inhibition [36].

Peptides were also successful for enamel remineralization. It is imperative to note that the acidic nature of dental cavities created by a massive amount of sugar intake leads to bacterial colonization and a reduction in the pH. Accordingly, the demineralization of the enamel surface begins [48]. In order to prevent this issue, numerous remineralizing agents were presented [48]. A perfect agent should be free of toxicity and qualified to initiate remineralization without any harm to the dental surface. Matrix-facilitated mineralization equal to a natural process should be carried out, though this ability is absent in almost all these agents [199]. The arrival of SAP P11-4 has overwhelmed this restriction. It has the ability to regenerate enamel. In addition, these agents initiate remineralization by making 3D constructions mimic the extracellular matrix of the dental surface [200]. Therefore, when talking about enamel remineralization, clinicians should focus on SAP due to its efficient and effective outcomes obtained in this review.

The occlusion of dentin tubules is considered possible with the help of peptides. This theory became conceivable when mineral particles were observed on dentinal tubules, thus reducing dentinal permeability and enhancing the seal of the material-tooth interfaces [57]. Bonding agents and desensitizers have been demonstrated to be effective for occluding tubules by mineral precipitation; however, these techniques are sensitive, and the long-term performance of the resin is doubtful [201,202]. As a balancing method for the protein mediation of hydroxyapatite mineralization, streamlined synthetic cationic macromolecules comprising poly(L-lysine) (PLL) that cover primary and secondary amine groups are organizationally comparable to the functional areas of the natural proteins and have further been presented to encourage silicification [203]. This review implies that this peptide-catalyst-mediated method of mineral formation for occluding tubules and/or reinforcing dentin-bonding resins might retain function on the dentin surface, advising a wide range of protective and treatment plans.

Peptides have also been successfully used as healthy promotors of the periodontium, acting as inflammatory mediators. Periodontitis is a chronic inflammatory and tissue-destructive illness. Meanwhile, the oral cavity with its polymicrobial effect makes it problematic to treat; thus, new healing approaches are mandatory. In a minimally invasive way, SAP delivers the benefit of being functional at a defect site without creating a toxic area [204]. Furthermore, their tunable mechanical characteristics and reasonably designed physicochemical features permit a high variety of encapsulated drugs [205]. Some peptides called P11-4 and P11-28/29 were considered SAP-applicable for periodontal therapy, due to their biocompatibility, injectability, tunable mechanical and physicochemical properties, and cargo-loading capacity [72].

Finally, peptides were used in the modification of dental materials such as dental adhesives and denture base resins. Recurrent decay that grows at the composite-tooth interface was demonstrated to be a disadvantage when using resin-based composite [163]. Primarily, the composite-tooth interface becomes coated by a low-viscosity adhesive system; however, when a fragile seal to the dentin is obtained, damage from enzymes, acids, and oral fluids will be achieved. This impairment is chief in crevices that are occupied by cariogenic bacteria such as *Streptococcus mutans* [206,207,208,209]. Various bacterial-inhibition strategies have been incorporated into adhesive systems, but none of these strategies address the multifaceted interplay of the mechanical and physicochemical influences of the durability of the adhesive seal at the composite-tooth interface. Antimicrobial peptides have been coupled into the adhesive system using non-bonded interactions [146], and subsequently, antimicrobial peptides were conjugated into the network of the adhesive system in order to improve the antimicrobials’ effectiveness [147]. An antimicrobial peptide AMP2-derivative (AMPM7) sequence using a functional spacer was used for integration into a monomer site. This adhesive system formed of co-tethered peptides demonstrated both localized calcium phosphate remineralization and strong metabolic inhibition of *S. mutans* [163]. An adhesive system incorporated with an antimicrobial peptide inhibited bacterial attack, and a hydroxyapatite-binding peptide promoted the remineralization of damaged tooth structures [146,163]. In 2017, Su et al. demonstrated that a cured antimicrobial peptide with nisin-incorporated dental adhesive showed a significant inhibitory effect on the growth of *S. mutans* [133], and recently, a paper showed that 3% (*w*/*v*) of nisin-incorporated universal adhesive system substantially inhibited the growth of both saliva-derived multispecies biofilms and *S. mutans* monospecific biofilms without hindering the bonding performance [166].

Moreover, it was demonstrated that *C. albicans* colonization on the denture’s base was significantly less than the control when histatin-adsorbed PMMA (poly methyl methacrylate), an antimicrobial peptide, was used [161]. Another report suggested that histidine-rich polypeptides were effective in the treatment of denture stomatitis [121], thus evidencing the important use of peptides in removable prostheses.

Some limitations relative to the applications of peptides in the dental field can be cited. One restriction is the absence of homogeneity of the type and obtention of the peptides used in the different applications described in the present review. Another limitation that can be highlighted is that due to the heterogeneity of the analytical techniques used for distinguishing the peptides, analyzing data using any statistical analysis was avoided.

## 5. Conclusions

The use of peptides has been gaining increasing attention in contemporary dentistry. Dental research evidence suggests that peptides have several applications, including osseointegration, guided tissue regeneration, vital pulp therapy, antimicrobial activity, enamel remineralization, and the surface modification of dental implants. The lack of clinical trials should be highlighted, leaving a wide space available for the investigation of peptides in dentistry.

## Figures and Tables

**Table 1:** Search strategy used in the MEDLINE database.

(Peptide) OR (Polypeptides) OR (Polypeptide) AND (Materials, Dental) OR (Dental Material) OR (Material, Dental)

**Table 2:** Characteristics of the included studies.

Study and Year	Type of Study	Peptide Used	Application	Main Results
Bagno, 2007 [41]	In vitro	Two adhesive peptides: an RGD-containing peptide and a peptide recorded on human vitronectin	Implant osseointegration	It was observed that there was a capacity of the peptides to promote enhanced cell adhesion
Artzi, 2006 [42]	Experimental study	A synthetic peptide (P-15)	Guided tissue regeneration and guided bone regeneration techniques	The use of a synthetic peptide showed increased osteoconductive and biocompatible features
Bröseler, 2020 [43]	Randomized clinical trial	Self-assembling peptide (SAP) P11-4	Early buccal carious lesions	Self-assembling peptide regenerated enamel caries lesions
Butz, 2011 [44]	Prospective in vivo study	Synthetic Peptide in a Sodium Hyaluronate Carrier (PepGen P-15 Putty)	Sinus grafting	The peptide evaluated was successful for maxillary sinus augmentation
Chung, 2013 [45]	In vitro	Asparagine–serine–serine (NSS) peptide.	Remineralization of eroded enamel.	Peptide increased the nanohardness and elastic modulus of eroded enamel
Altankhishig, 2021 [46]	In vitro and in vivo	Peptide	Vital pulp therapy	The dentin phosphophoryn-derived arginine-glycine-aspartic acid-containing peptide showed adequate properties as a bioactive material for dentin regeneration
Afami, 2021 [47]	In vitro	Ultrashort peptide hydrogel, (naphthalene-2-ly)-acetyl-diphenylalanine-dilysine-OH (NapFFεKεK-OH)	Antimicrobial activity and angiogenic growth factor release by dental pulp stem/stromal cells	Peptide-containing hydrogels have potential in tissue engineering for pulp regeneration
Babaji, 2019 [48]	In vitro	SAP P11-4 and casein phosphopeptides-amorphous calcium phosphate (CPP-ACP)	Enamel remineralization	The peptide was more effective and efficient when compared to CPP-ACP
Dettin, 2002 [49]	In vitro	Novel osteoblast-adhesive peptides	Osteoblast adhesion	The novel peptide promotes proteoglycan-mediated osteoblast adhesion efficiently
Cirera, 2019 [50]	In vivo	TGF-β1 inhibitor peptide: P144	Osseointegration of synthetic bone grafts	The healing period of osseointegrated biomaterials can be shortened when peptide biofunctionalization is used
Boda, 2020 [51]	In vitro	Mineralized nanofiber segments combined with calcium-binding bone morphogenetic protein 2 (BMP-2)-mimicking peptides	Alveolar bone regeneration	Mineralized nanofibers functionalized with peptides have the potential to regenerate craniofacial bone defects
Chen, 2017 [52]	In vivo	GL13K-peptide	Osseointegration of implants	This study showed that titanium dental implants with an antimicrobial GL13K peptide coating enables in vivo implant osseointegration
Aref, 2022 [53]	In vitro	CPP-ACP	White spot lesion	CPP-ACP could be a promising approach to manage WSLs efficiently, with subsequent universal adhesive resin infiltration
Aruna, 2015 [54]	Clinical	Gingival crevicular fluid (GCF) N-terminal telopeptides of type I collagen (NTx)	Periodontal therapy	Cross-linked NTx can be successfully estimated in the GCF of chronic periodontitis subjects
Brunton, 2013 [55]	A clinical trial	Biomimetic SAP: P11-4	Early caries lesions	Treatment of early caries lesions with P11-4 is safe, and a single application of this peptide is associated with significant enamel regeneration
Fang, 2020 [56]	In vitro	Two hexapeptide coatings	Dental implants	The novel hexapeptide coating can inhibit the attachment of Porphyromonas gingivalis and prevent the formation of dental biofilm
Goldberg, 2009 [57]	In vitro	Polypeptide	Occluding dentin tubules	Peptide catalysts that mediate mineral formation can retain functionality on dentin, suggesting a wide range of preventive and treatment strategies
Amin, 2012 [58]	In vitro	Amelogenin Peptides	Osteogenic differentiation	Amelogenin-derived peptide could be a useful tool for limiting pathological bone cell growth
Godoy-Gallardo, 2015 [59]	In vitro	hLf1-11 Peptide	Antibacterial properties on titanium surfaces	A greater amount of peptide attached to the surfaces functionalized via atom transfer radical polymerization than those functionalized via silane
Dommisch, 2019 [60]	In vivo and in vitro	Antimicrobial peptides	Gingival inflammation	The study delivers evidence on the role of antimicrobial peptides as guardians of a healthy periodontium
Dommisch, 2015 [61]	Experimental study	Antimicrobial peptides	Gingivitis	Differential temporal expression for antimicrobial peptides could guarantee continuous antimicrobial activity alongside changes in the bacterial composition of the growing dental biofilm
Fernandez-Garcia, 2015 [62]	In vitro	Peptide-functionalized zirconia	Implant	Surface bioactivation of zirconia-containing constituents for dental implant applications will allow their perfected clinical implementation by incorporating signaling oligopeptides to accelerate osseointegration, improve mucosal sealing, and/or incorporate antimicrobial properties to avoid peri-implant infections
Fiorellini, 2016 [63]	In vitro	Osteopontin-derived synthetic peptide: OC-1016	Osseointegration of implants	OC-1016 was capable of meaningfully accelerating the initial stage of osseointegration and bone healing around implants
Goeke, 2018 [64]	Clinical	Antimicrobial peptides	Caries risk	The incidence of low-susceptible strains to antimicrobial peptides appears to relate to individual caries status
Galler, 2012 [65]	In vitro	SAP hydrogel	Dental pulp tissue engineering	The use of this innovative biomaterial was considered a highly favorable candidate for upcoming treatment hypotheses in regenerative endodontics
Kirkham, 2007 [66]	In situ	SAP scaffolds	Enamel remineralization	SAP might be useful for dental tissue engineering
Kämmerer, 2011 [67]	In vitro	RGD peptides	Dental implants	Modifications of titanium surfaces with c-RGD peptides are an encouraging way to endorse endothelial cell growth
Golland, 2017 [68]	In vitro	SAP	Remineralization of white spot lesions	The application of SAP on demineralized bovine enamel indicated an irregular crystal or a lack of remineralization
Hsu, 2010 [69]	In vitro	Aspartate-serine-serine (8DSS) pep- tides	Nucleation of calcium phosphate carbonate from free ions	8DSS peptides reduced the surface roughness of demineralized enamel and promoted the uniform deposition of nano-crystalline calcium phosphate carbonate over demineralized enamel surfaces
Kwak, 2017 [70]	In vitro	Leucine-rich amelogenin peptide (LRAP)	Enamel regeneration	LRAP has the power to enhance the linear growth of mature enamel crystals
Kong, 2015 [71]	In vivo	Histatin-5 (Hst-5)	Oral Candidiasis	Hst-5 was able to clear existing lesions
Koch, 2019 [72]	In vitro	SAP: P11-4 and P11-28/29	Periodontal therapy	SAP hydrogels were effective for periodontal therapy
Hashimoto, 2011 [73]	In vitro	Peptide motif	Zirconia	A peptide motif was successful in binding zirconia
Kind, 2017 [74]	In vitro	SAP: P11-4	Remineralization of carious lesions	The application of P11-4 might facilitate the subsurface regeneration of the enamel lesion
Gonçalves, 2020 [75]	In vitro	Casein phosphopeptide-amorphous calcium phosphate (MI Paste Plus)	Enamel demineralization and dental caries	MI Paste Plus might be effective in improving oral health
Kim, 2019 [76]	In vitro and in vivo	A laminin-derived functional peptide	Implant	Peptide DN3 promotes bone healing
Kohgo, 2011 [77]	In vitro	SAP	Implant	SAP could be useful for bone regeneration around dental implants
Gungormus, 2012 [78]	Ex vivo	Amelogenin-derived peptides	Periodontal tissues	Amelogenin-derived peptide 5 promoted the regeneration of periodontal tissues
Kakegawa, 2010 [79]	In vitro	Enamel sheath protein peptides	Construction of the enamel sheath during tooth development	A specific peptide sequence encourages the cytodifferentiation and mineralization activity of human periodontal ligaments
Kramer, 2009 [80]	In vitro	Integrin blocking peptide	Titanium surfaces	Antibody and peptide treatment reduced the number of fibroblast cells involved on the implant surfaces
Hua, 2010 [81]	In vitro	Antimicrobial peptide	Oral cavity	The antimicrobial peptide was demonstrated as an anti-Candida agent
Hua, 2010 [81]	In vitro	Antimicrobial peptide	Oral cavity	The antimicrobial peptide exhibits potent activity against both *A. actinomycetemcomitans* and *P. gingivalis* biofilms
Kohlgraf, 2010 [82]	In vitro	Human neutrophil peptide α-defensins (HNPs)	Cytokine responses	The ability of HNPs to attenuate proinflammatory cytokines was dependent upon both the defensin and antigen of *P. gingivalis*
Holmberg, 2013 [83]	In vitro	Antimicrobial peptide: GL13K	Dental and orthopedic implants	The antimicrobial activity and cytocompatibility of GL13K-biofunctionalized titanium make it a promising candidate for sustained inhibition of bacterial biofilm growth
Koidou, 2019 [84]	In vitro	Bioinspired peptide coatings	Peri-implant mucosal Seal	Peptide coatings were considered a promising candidate for inducing a peri-mucosal seal around dental implants
Knaup, 2021 [85]	In vitro	SAP: P11-4	Metal brackets	The application of the caries-protective SAP P11-4 before the bonding of brackets did not influence the shear bond strength
Kihara, 2018 [86]	In vitro	Novel synthetic peptide (A10)	Titanium surface	The novel peptide has a useful presentation that might enhance advanced clinical outcomes by means of titanium implants and abutments by preventing or reducing peri-implant disease
Jablonski-Momeni, 2020 [87]	In vitro	SAP P11-4	Early enamel lesions adjacent to orthodontic brackets	The application of p11-4 with fluoride varnish was demonstrated to be superior for the remineralization of enamel adjacent to brackets when compared to the use of fluorides alone
Kamal, 2018 [88]	In vitro	SAP P11-4	Artificially induced enamel lesions	SAP confers a higher remineralizing efficacy
Mao, 2021 [89]	In vitro	CPP-ACP	Dental caries	The use of 5% CPP-ACP reduced 39% of bacterial biofilm
Makihira, 2011 [90]	In vivo	Antimicrobial peptide derived from histatin: JH8194	Dental implant	JH8194 might deliver a viable biological modification of titanium surfaces to amplify trabecular bone formation around dental implants
Li, 2014 [91]	In vitro	Synthetic and self-assembled oligopeptide amphiphile (OPA)	Mineralization of enamel	OPA was successful in the biomimetic mineralization of demineralized enamel
Liu, 2016 [92]	Experimental	Chimeric peptides comprising antimicrobial and titanium-binding motifs	Biofilm formation	Chimeric peptides provide a promising alternative to inhibit the formation of biofilms on titanium surfaces with the power to prevent peri-implantitis
Min, 2013 [93]	In vitro	Laminin-derived functional peptide, Ln2-P3	Implant	An Ln2-P3-coated implant surface enhances bone cell adhesion
Moore, 2015 [94]	Ex vivo	Multidomain peptide hydrogels	Dental pulp	Multidomain peptide hydrogels offered centrally and peripherally within whole dental pulp tissue are demonstrated to be biocompatible and preserve the architecture of the local tissue
Muruve, 2017 [95]	In vitro	PEGylated metal-binding peptide (D-K122-4-PEG)	Titanium surface	D-K122-4-PEG promotes resistance to corrosion
Nguyen, 2018 [96]	In vitro	Dentinogenic peptide	Dental pulp stem cells	The SAP promised guided dentinogenesis
Mardas, 2007 [97]	An experimental study in rats	PepGen	Bone regeneration	The anorganic bovine-derived hydroxyapatite matrix coupled with a synthetic cell-binding peptide failed to promote new bone formation
Mateescu, 2015 [98]	In vitro	Antimicrobial peptide Cateslytin	Peri-implant diseases	The new peptide could be ideal in the prevention of peri-implant diseases
Liu, 2021 [99]	In vitro	RADA16-I: (SAP)	Pulp regeneration	The novel SAP could be ideal in endodontic tissue engineering
Li, 2020 [100]	In vitro	GH12: antimicrobial peptide	Root canal irrigant	GH12 suppressed *E. faecali* in dentinal tubules
Mancino, 2022 [101]	In vitro	Catestatin-derived peptides	Oral candidiasis	The catestatin-derived peptides were considered for the treatment of oral candidiasis
Mai, 2016 [102]	In vitro	Antimicrobial peptides	Caries and pulpal infections	Antimicrobial peptide mimics offer opportunities for new therapeutics in regenerative endodontics and root canal treatments
Lv, 2015 [103]	In vitro	Amelogenin based peptide	Remineralization of initial enamel caries	The amelogenin-based peptide enhances enamel caries remineralization
Lee, 2007 [104]	In vitro and in vivo	Collagen-binding peptide	Osteogenesis	Collagen-binding peptide induced biomineralization of bone
Liang, 2018 [105]	In vitro	8DSS peptide	Dentinal tubule occlusion	8DSS peptide induced strong dentinal tubule occlusion and can be used in dentin hypersensitivity
Lee, 2018 [106]	In vitro and in vivo	Bone formation peptide-1 (BFP1)	Bone regeneration	BFP1 was considered promising for bone repair
Na, 2005 [107]	Preformulation study	Antimicrobial decapeptide (KSL)	Antiplaque agent	KSL served as a novel antiplaque agent in the oral cavity
Magalhães, 2022 [108]	In vitro	Self-assembly peptide: P11-4	Bleached enamel	The use of P11-4 after bleaching results in the fastest recovery to baseline enamel properties
Lallier, 2003 [109]	In vitro	Collagen-binding peptide P-15	Periodontal treatment	P-15 promoted fibroblast attachment to root surfaces
Li, 2021 [110]	In vitro	Small-size peptide: RR9	Oral streptococci	RR9 might be considered a possible antimicrobial agent for periodontal disease
Matsugishi, 2021 [111]	In vitro	Rice peptide	Biofilm formation	Rice peptide hindered the biofilm formation of *F. nucleatum* and *P. gingivalis*
Li, 2022 [112]	In vitro	Amelogenin-based peptide hydrogel	Human dental pulp cells	The amelogenin peptide hydrogel enhanced mineralization and encouraged odontogenic differentiation
Mishra, 2019 [113]	A randomized clinical trial	Anorganic bone matrix/cell-binding peptide (ABM/P-15)	Human infrabony periodontal defects	The combination of ABM/P-15 was established to be a favorable material for periodontal regeneration
Padovano, 2015 [114]	In vitro	DMP1-derived peptides	Remineralization of human dentin	DMP1-derived peptides could be useful in modulating mineral deposition
Park, 2020 [115]	In vitro	BMP-mimetic peptide	Dental pulp stem cells	BMP-mimetic peptide accelerated human dental pulp stem cells
Pellissari, 2021 [116]	In vitro	Statherin-derived peptides	Biofilm development	The natural peptides from statherin are able to decrease biofilm proliferation and Candida albicans colonization
Petzold, 2012 [117]	In vivo	Proline-rich synthetic peptide	Titanium implants	Proline-rich peptides have a probable biocompatible capacity for endorsing osseointegration by lessening bone resorption
Picker, 2014 [118]	In vitro	Binding peptides	Calcium silicate hydrate	A new strong calcium silicate hydrate-binding additive influenced the physical properties of cement
Pihl, 2021 [119]	In vivo	Antimicrobial peptide: RRP9W4N	Titania implant	RRP9W4N was demonstrated to be successful in the control of infection in osseointegrating implants
Ren, 2018 [120]	In vitro	Chitosan hydrogel containing amelogenin-derived peptide	Initial caries lesions	Chitosan hydrogel containing amelogenin-derived peptide was demonstrated to be effective in controlling caries and promoting the remineralization of the initial enamel carious lesion
Santarpia, 1991 [121]	In vivo	Histidine-rich polypeptides	Denture stomatitis	Histidine-rich polypeptides were effective in the treatment of denture stomatitis
Schmidlin, 2015 [122]	In vitro	SAP	Mineralization of artificial caries lesions	SAP improved the hardness profile of deep demineralized artificial lesions
Schmitt, 2016 [123]	In vivo	Synthetic peptide (P-15)	Osseointegration	There is no advantage in the early phase of osseointegration for dental implants with P-15-containing surfaces
Schuler, 2006 [124]	In vitro	RGDSP-peptide sequence	Titanium dental implant	There is no communication between RGD-peptide surface density and surface topography for osteoblasts
Schuster, 2020 [125]	In vitro	Hydroxyapatite/BMP-2 mimetic peptide	Bone tissue engineering	Biofunctionalization of collagen-hydroxyapatite composites with BMP-2 simulated peptides was considered cost-effective and fast for prolonged and improved jaw periosteal cell proliferation
Secchi, 2007 [126]	In vitro	Arginine-glycine-aspartic acid (RGDS) peptides	Implant	The modification of the titanium surface with RGDS peptides promoted osseointegration
Segvich, 2009 [127]	In vitro	Binding peptide sequences	Bone regeneration	The binding peptide sequences can be used in dentin and bone tissue engineering
Sfeir, 2014 [128]	In vitro	Multiphosphorylated peptides	Mineralized collagen fibrils of bone and dentin	Using phosphopeptides, there is progress in biomimetic nanostructured materials for mineralized tissue regeneration and repair
Shi, 2015 [129]	In vitro	Antimicrobial peptide-loaded coatings	Dental implant	The antimicrobial peptide-loaded coatings were demonstrated to be a potential approach for preventing peri-implantitis
Shinkai, 2010 [130]	In vitro	Synthetic peptides derived from dentin matrix protein 1 (pA and pB)	Direct pulp capping and bonding agent	The primer containing synthetic peptides derived from dentin matrix protein 1 negatively affected the bond strength to dentin
Shinkai, 2010 [131]	In vitro	Synthetic peptides (pA and pB)	Bonding agent	A significant difference was seen in bond strength among CaCl_2_ concentrations in Primer-I (comprising 10 wt.% CaCl_2_) and pA/pB concentrations in Primer-II comprising 10 wt.% pA/pB, and there is a noteworthy interaction between these two factors
Shuturminska, 2017 [132]	In vitro	Statherin-derived peptide	Enamel biomineralization	The use of statherin-derived peptide was considered effective in enamel therapy
Su, 2017 [133]	In vitro	Peptide nisin	Dental adhesive	The cured nisin included in the dental adhesive showed a noteworthy inhibitory effect on the growth of *S. mutans*
Suaid, 2010 [134]	Histologic and histomorphometric study	Anorganic bone matrix–synthetic cell-binding peptide 15	Periodontal class III furcation defects	The use of anorganic bone matrix–synthetic cell-binding peptide 15 was effective in bone formation
Sugawara, 2016 [135]	In vitro	Platelet-activating peptide	Titanium surface	An epithelial basement membrane was formed on the titanium surface when platelet activating peptide was used
Sun, 2016 [136]	Clinical	Peptidome	Early childhood caries	The magnetic bead-founded matrix-assisted laser desorption/ionization time-of-flight mass spectrometry was considered an effective technique for screening distinctive peptides from the saliva of junior patients with early childhood caries
Takahashi, 2002 [137]	In vitro	Dipeptide: aspartylaspartate and glutamylglutamate	Periodontal pathogens	Dipeptides can be employed as growth substrates for *P. intermedia*, *P. gingivalis*, *F. nucleatum*, and *P. nigrescens*
Tanhaieian, 2020 [138]	In vitro	Recombinant peptide	Dental diseases	The recombinant peptide was demonstrated effective as an antimicrobial agent against *E. faecalis* and oral streptococci
Üstuün, 2019 [139]	In vitro	SAP: P11-4	Artificial enamel lesions	P11-4 was demonstrated to have the best remineralization efficacy
Wag, 2020 [140]	In vivo	Neural peptide	Angiogenesis and osteogenesis around oral implants	Alpha-calcitonin gene-related peptide up-regulated the expression of Hippo-YAP and downstream genes in order to encourage osteogenesis and angiogenesis around the implants
Wang, 2015 [141]	In vitro	Peptide DJK-5	Dentin canals	The peptide DJK-5 showed an imperative antibacterial property against mono- and multispecies biofilms in dentin canals
Warnke, 2013 [142]	In vitro	Human beta-defensins (HBDs), small cationic antimicrobial peptides	Dental implants	HBD-2 is not only biocompatible with but further encourages the proliferation of human mesenchymal stem cells
Wener, 2009 [143]	In vitro	Laminin-derived peptide	Dental implants	Laminin-derived peptide improved and enhanced the integration of soft tissue on titanium implants used in dentistry
Winfred, 2014 [144]	In vitro	Cationic peptides	Endodontic procedures	Cationic peptides prevented the spread of endodontic infections
Wu, 2022 [145]	In vitro	TGF-β1 binding peptide–modified bioglass	Endodontic therapy	TGF-β1 binding peptide–modified bioglass was effective for regeneration in endodontic therapy
Xue Xie, 2019 [146]	In vitro	Antimicrobial peptide	Dental adhesive system	Antimicrobial peptide-hydrophilic adhesive delivers an advanced adhesive/dentin interface
Xue Xie, 2020 [147]	In vitro	Antimicrobial peptide	Dental adhesive system	Peptide-conjugated dentin adhesives were effective in secondary caries treatment and improved the durability of dental composites
Yakufu, 2020 [147]	In vitro	Osteogenic growth peptide (OGP)	Osteogenesis activity	OGP was promising in dental and orthopedic applications
Yamamoto, 2012 [148]	In vivo	Peptide including Arg-Gly-Asp (RGD) sequence	Periodontal ligament cells	Glial cell line-derived neurotrophic factor, which was hindered by pre-treatment with the peptide-embracing Arg-Gly-Asp (RGD) sequence, enhanced the appearance of bone sialoprotein and fibronectin on human periodontal ligament cells
Yamashita, 2010 [149]	In vitro	Anabolic peptide	Periodontal regeneration	Anabolic peptide has a positive influence on bone cells
Yang, 2017 [150]	In vitro	Peptide-modified tannic acid	Hydroxyapatite surface	Peptide-modified tannic acid inhibited the adhesion of bacteria
Yang, 2018 [151]	In vitro	Salivary acquired pellicle (SAPe)-inspired peptide DDDEEK	Biofilms	SAPe-inspired peptide DDDEEK has a great advantage in the field of implant materials
Yang, 2017 [152]	In vitro and in vivo	Bioinspired peptide-decorated tannic acid	Remineralization of tooth enamel	Bioinspired peptide-decorated tannic acid has a good influence on the remineralization of tooth enamel
Yang, 2019 [153]	In vitro	Dual-functional polypeptide	Implant materials	Dual-functional polypeptide has a potential application in the treatment of hard tissue-related diseases
Yang, 2019 (b) [154]	In vitro and in vivo	Immunomodulatory peptide 1018	Plaque biofilms	Immunomodulatory peptide 1018 was effective as an anti-biofilm agent
Yang, 2017 (b) [155]	In vitro	DpSpSEEKC peptide	Demineralized tooth enamel	DpSpSEEKC restored demineralized tooth enamel
Yang, 2020 [156]	In vitro	Cell-adhesion peptides via polydopamine crosslinking	Zirconia abutment surfaces	Cell-adhesion peptides improved soft tissue integration around zirconia abutments via polydopamine crosslinking
Yazici, 2013 [157]	In vitro	Modular peptides	Titanium implant	Modular peptides on titanium surfaces improved the bioactivity of fibroblast and osteoblast cells on implant-grade materials
Ye, 2017 [158]	In vitro	Peptide-based approach	Adhesive-dentin interface	The peptide-based remineralization approach was effective in designing integrated tissue-biomaterial interfaces
Ye, 2019 [159]	In vitro	*D-enantiomeric* and *L-enantiomeric* antimicrobial peptides	Root canal wall biofilms	*D-enantiomeric* peptides exhibited more antimicrobial potent activity than *L-enantiomeric* peptides against *E. faecalis* biofilms on the canal space
Yonehara, 1986 [160]	In vivo	Opioids and opioid peptides	Tooth pulp stimulation	There is an interaction between substance P and enkephalin systems in the superficial layer of the brain-stem trigeminal sensory nuclear complex for the regulation of dental pain transmission. In addition, the native application of naloxone (5 × 10^−7^ M) partly antagonized the inhibitory effects of locally applied morphine and the opioid peptide
Yoshinari, 2005 [161]	In vitro	Antimicrobial peptide histatin 5	Poly (methyl methacrylate) denture base	*C. albicans* colonization on histatin-adsorbed PMMA was knowingly less than the control
Yoshinari, 2010 [162]	In vitro	Antimicrobial and titanium-binding peptides	Titanium surfaces	Antimicrobial and titanium-binding peptides were encouraging for the reduction of biofilm formation on titanium surfaces
Yuca, 2021 [163]	In vitro	Dual-peptide tethered polymer system	Dental adhesives	The adhesive system formed of co-tethered peptides revealed both localized calcium phosphate remineralization and strong metabolic inhibition of *S. mutans*
Zhang, 2022 [164]	In-vitro	Dual-sensitive antibacterial peptide	Dental caries	This peptide prevented damage from bacteria and, thus, from dental caries
Zhang, 2016 [165]	In vitro	D-Enantiomeric peptide	Oral biofilms	D-enantiomeric peptide was effective against oral biofilms
Zhao, 2020 [166]	In vitro	Antimicrobial peptide nisin	Dental adhesive	3% (*w*/*v*) nisin-incorporated Single Bond Universal substantially inhibited the development of both saliva-derived multispecies biofilms and monospecific *S. mutans* biofilms without hindering the bonding performance
Zhou, 2008 [167]	In vitro	Genetically engineered peptides for inorganics	Tooth repair	Genetically engineered peptides for inorganics were effective in tooth repair
Zhou, 2015 [168]	In vitro	Antimicrobial peptide	Titanium surfaces	Antimicrobial peptide provided a promising bifunctional surface
Gungormus, 2021 [169]	In vitro	Peptide-assisted pre-bonding	Remineralization of dentin	Pre-bonding remineralization of dentin using peptide during 10 min notably enhanced the stiffness of dentin and the resistance to hydrolysis. In addition, it can reduce shrinkage due to drying
Koidou, 2018 [84]	In vitro	Laminin 332- and ameloblastin-derived peptides (Lam, Ambn)	Peri-implant mucosal seal	Laminin 332- and ameloblastin-derived peptides were demonstrated to be effective in producing a peri-mucosal seal around dental implants
Gug, 2022 [170]	In vivo	CPNE7-derived functional peptide	Dentin regeneration of dental caries	CPNE7-derived functional peptide repaired caries by dentin regeneration

## Data Availability

The data presented in this study are available on reasonable request from the first author (L.H.).
